# Immunological Effects of Recombinant *Lactobacillus casei* Expressing IHNV G Protein and Rainbow Trout (*Oncorhynchus mykiss*) Chemokine CK6 as an Oral Vaccine

**DOI:** 10.3389/fimmu.2022.927443

**Published:** 2022-06-16

**Authors:** Jinhui Sun, Mengmeng Zhang, Dandan Zhao, Jiawei Yang, Yanxue Shi, Baoxing Xu, Xuefei Liu, Xueting Guan, Wen Shi, Min Liu

**Affiliations:** College of animal Science and Technology, Northeast Agicultural University, Harbin, China

**Keywords:** IHNV, G protein, CK6, *Lactobacillus casei*, oral vaccines

## Abstract

IHNV is a virus that infects salmonids and causes serious economic damage to the salmonid farming industry. There is no specific treatment for the disease caused by this pathogen and the main preventive measure is vaccination, but this is only possible for small groups of individuals. Therefore, it is important to investigate new oral vaccines to prevent IHNV. In this study, the CK6 chemokine protein of rainbow trout and the truncated G protein of IHNV were used to construct a secretory expression recombinant *L.casei* vaccine for rainbow trout. The results showed that the levels of IgM and IgT antibodies in rainbow trout reached the highest level on the 15th day after the secondary immunization, and the antibodies exhibited high inhibitory activity against viral infection. Furthermore, the expression of relevant cytokines in different tissues was detected and found to be significantly higher in the oral vaccine group than in the control group. It was also found that pPG-612-CK6-G/*L.casei* 393 could stimulate splenic lymphocyte proliferation and improve mucosal immunity with significant differences between the immunized and control groups. When infected with IHNV, the protection rate of pPG-612-CK6-G/*L.casei* 393 was 66.67% higher than that of the control group. We found that pPG-612-CK6-G/*L.casei* 393 expressed and secreted the rainbow trout chemokine CK6 protein and IHNV truncated G protein, retaining the original immunogenicity of rainbow trout while enhancing their survival rate. This indicates that recombinant *L.casei* provides a theoretical basis and rationale for the development of an oral vaccine against IHNV and has important practical implications for the protection of rainbow trout from IHNV infection.

## Introduction

Infectious haematopoietic necrosis (IHN) is a viral disease that is highly infectious to salmonids (1). The disease has spread widely around the world and caused outbreaks, leading to huge economic losses to the salmon farming industry (2). The pathogen which causes IHN is IHNV, which is an antisense single-stranded RNA ejection virus with extremely high morbidity and lethality (3–6). IHNV consists of six proteins, P, G, M, N, L and NV respectively (7–10). The main approach for the treatment of this disease is prevention, supplemented by drug therapy. Over time, some problems have been revealed, such as drug residues and heavy metals. Therefore, as an alternative to drugs, the development of vaccines has become a new effective way to prevent and treat this disease. Anderson et al. used chemical inactivation of IHNV to create vaccines for the immunization of rainbow trout, and found that a propionolactone inactivated whole virus vaccine provided the fish with significant protection (11). M. Adomako et al. created DNA vaccines made from IHNV encapsulated with PLGA nanoparticles and was found to prevent outbreaks of IHN after immunizing rainbow trout orally (12). The IHNV vaccine is currently in the research phase and only one vaccine has been commercialized for use (13). Given the economic impact of IHNV, the development of a vaccine is an issue of substantial importance that needs to be addressed quickly.

The role of G proteins is to stimulate the production of antibodies in the host. The G protein of IHNV is a structural protein and also an important transmembrane protein of the virus (14). The G protein is the major antigenic cluster of IHNV and is involved in viral adsorption, endocytosis and replication (15, 16). The extra-membrane region of the IHNV G protein is the main region that affects the antigenic properties and biological characteristics of IHNV. Changes in amino acids at specific locations within the extra-membrane region can alter the virulence and antigenic properties of the virus (17–19). It has been reported that the pcDNA 3.1 eukaryotic expression vector has been combined with the IHNV G protein to form a recombinant expression vector to immunizse rainbow trout, and it was found that the nucleic acid vaccine provided up to 90% protection against rainbow trout (20). Anderson et al. developed a DNA vaccine by recombining the G protein gene of IHNV with a plasmid containing a cytomegalovirus promoter and found that the immune protection of this vaccine could reach 85-98% (21). From these studies it can be seen that the G protein plays an important role in the development of IHNV vaccines.

*Lactobacillus* is a Gram-positive bacterium that is very common in humans and animals. It is not pathogenic to the organism and protects against viruses. It is also a good vector for the expression and delivery of exogenous proteins and is widely used in the development of oral and lactic acid bacteria vaccines (22, 23). It has been found that using *Lactobacillus* as a carrier can enhance the immune system and prevent infection (24). It was shown that recombinant *lactobacilli* constructed by combining the S protein of TGEV with *L.casei* were effective at immunizing mice. It was found that antibody levels in the mice after vaccination were elevated, indicating that the recombinant *Lactobacillus* were able to induce local and systemic immune responses in immunized mice (25). In another study researchers immunised mice with recombinant *Lactobacillus* constructs with VP8 from *Rotaviruses* and showed that the mice exhibited significant intestinal mucosal antibody protection up to 50% (26). In aquatic vaccine studies, it was found that *Lactobacillus* could also act as a vehicle to deliver viral antigens and induce faster specific immunity in fish (27).

In this study, a recombinant plasmid was constructed by truncating the IHNV G gene with the chemokine CK6 gene of rainbow trout. The recombinant plasmid was introduced into *L.casei* using electroconversion to construct a recombinant *L.casei* expression system named pPG-612-CK6-G/*L.casei* 393. The immunoprotective effect of the vaccine was evaluated by testing the immunogenicity and protection of the recombinant *L.casei* expression system. This provided a theoretical basis for the subsequent development of a vaccine against infectious haematopoietic organ necrosis.

## Materials and Methods

### Growth Conditions for Cells, Viruses, Bacteria

The IHN virus was inoculated into CHSE-214 cells and cultured at 18°C. The CHSE-214 cells were inoculated in L-15 containing 10% FBS and cultured at 22°C. *Lactobacillus* and *E.coli* TG1 were inoculated in MRS and LB medium respectively and cultured at 37°C. The concentration of antibiotics used was chloramphenicol (Cm) at 10 μg/mL and sodium ampicillin at 100 μg/mL. The strains and plasmids used in this study are listed in **Table 1**.

**Table 1:** Strains and plasmids used in this study.

Strain or plasmid	Phenotype	Source or reference
Plasmid		
pPG-612	(Cm^R^)	Provided by NIZO Institute, Netherlands
pMD19-T-simple	(Amp^R^)	TaKaRa
Strains		
*L.casei* ATCC393		Provided by NIZO Institute, Netherlands
*E.coli* TG1		This study
*E.coli* DE3		This study

### Experimental Fish

Rainbow trout weighing 10 ± 0.5 g were bought from the Bohai Coldwater Fish Experiment Station, Heilongjiang Institute of Fisheries Science (Mudanjiang, China). Healthy rainbow trout were temporarily reared for one week in a laboratory at a pathogen-free water temperature of 18°C and randomly tested for viruses. The rainbow trout were grouped according to experimental needs. All experimental fish were kept in accordance with animal welfare standards.

### Construction of Recombinant Plasmid pPG-612-CK6-G

The *E.coli* TG1 containing pMD18-T-CK6 plasmid was inoculated in LB and incubated at 37°C. Plasmids were extracted according to the instructions of the extraction kit. RNA was extracted from the viral liquid using the Trizol method and then reverse transcribed into cDNA. The CK6 gene was amplified using primers C1 and C2, and the G gene was amplified by primers C3 and C4. The target gene was linked to the vector pMD19-T-simple. and the G gene was cleaved with *Bam*H I and *Xho* I, while the CK6 gene was cleaved with *Sph* I and *Bam*H I. The recombinant plasmid pPG-612-CK6-G was constructed by ligating the digested target gene to the pPG-612 vector. The pPG-612-CK6-G was transferred into *E.coli* TG1 and identified. The primers for PCR used in this paper are listed in **Table 2**. Enzyme cut sites are indicated by underlining.

**Table 2:** Primers for PCR and qRT-PCR.

Primer	Nucleic acid sequence	Primer	Nucleic acid sequence
C1	GCATGCCGTGTGTCTGACACGGGTCGTATGA	EF-1α-F	CAAGGATATCCGTCGTGGCA
C2	GGATCCGGATCATCGGGTCAGCAGCTGCATGTTGAAAATGTTC	EF-1α-R	ACAGCGAAACGACCAAGAGG
C3	CGCGGATCCATGCTCGGTGACCTGATA	IFN-1-F	ACCAGATGGGAGGAGATATCACA
C4	CCGCTCGAGCCAGAAACTCCAGTG	IFN-1-R	GTCCTCAAACTCAGCATCATCTATGT
MX1-F	AGCTCAAACGCCTGATGAAG	IRF1-F	AGGCTAATTTCCGCTGTGCA
MX1-R	ACCCCACTGAAACACACCTG	IRF1-R	TTTTGTAGACGCGCACTGCT
IRF3-F	TGGACCAATCAGGAGCGAAC	IL-1β-F	GCAGCTCCATAGCCTCATT
IRF3-R	AGCCCACGCCTTGAAAATAA	IL-1β-R	TGTTGGAGTTGGAGTCGGCG
IRF7-F	GAGGAGTGGGCAGAGAACTA	TNF-α-F	AGCATGGAAGACCGTCAACGAT
IRF7-R	TTCTGGGAGACTGGCTGGG	TNF-α-R	ACCCTCTAAATGGATGGCTGCTT
IL-8-F	ACCATTACTGAGGGGATGAGTCTGA	IL-6-R	CCTCAGCAACCTTCATCTGGTC
IL-8-R	CATCTCCACCTTCTTAATGAGCCTA	IL-6-F	CCTTGCGGAACCAACAGTTTG

### Construction and Identification of pPG-612-CK6-G/*L.casei* 393

*L.casei* was cultured in MRS medium containing 2% glycine at 37°C until OD_600_ was 0.4-0.8, and it was treated with receptor cells in EPWB and EPB solution. We used the following procedure: 1 μL of rpPG-612 was mixed well with 200 μL receptor cells, then chilled in an ice bath for 1 min and added to the electroshock cup. After the electroshock 1 mL of non-resistant medium was then added mixed well before incubating at 37°C for 2 h. Then 200 μL of bacterial solution was taken and coated in resistant MRS solid medium and incubated overnight at 37°C. The colonies from the solid plate were picked for PCR identification. The correctly identified pPG-612-CK6-G/*L.casei* 393 was inoculated into MRS resistance medium and incubated until OD_600_ was 0.6-0.8. The bacteria were inoculated at a ratio of 1:10 in a new MRS resistance medium containing 2% xylose for 20 hours to induce. The induced organisms were taken and treated with lysozyme to obtain the protein and identified.

### The Genetic Stability and Growth Curve of pPG-612-CK6-G/*L.casei* 393

The correctly transformed pPG-612-CK6-G/*L.casei* 393 was inoculated as the first generation at a ratio of 1:100 into MRS medium overnight at 37°C. The pPG-612-CK6-G/*L.casei* 393 was continuously passaged to the 50th generation and the bacterial solutions were taken every five generations for identification. The 50th generation of pPG-612-CK6-G/*L.casei* 393 was inoculated at a ratio of 1:100 in new MRS medium containing Cm^+^ and incubated at 37°C. The bacterial solution was taken every 4 hours and OD_600_ was measured for 36 h. The growth curve was drawn according to the OD_600_.

### Detection of Specific Antibodies in Serum, Skin and Intestinal Mucus

The pPG-612-CK6-G/*L.casei* 393 was inoculated into MRS medium and incubated at 37°C until OD_600_ was 0.6-0.8. Bacterial broth was inoculated at a ratio of 1:10 in new MRS medium containing 2% xylose to induce. The induced bacteria were washed with PBS and adjusted to a concentration of 1 × 10^10^ CFU/mL (OD_600_ = 1.0). The adjusted bacterial solution was used to immunize rainbow trout by oral injection. The experimental group was orally given 200 μL of pPG-612-CK6-G/*L.casei* 393 suspension, the control group was orally given pPG-612/*L.casei* and the blank group was orally given phosphate buffered solution (PBS). The second immunization was performed on the 30th day after the first immunization. On days 1, 15, 30, 45 and 60 after the second immunization, blood, body surface and intestinal mucus were taken, processed and tested for specific antibodies using indirect ELISA. At the same time, the brain, kidney, muscle, spleen, gill and intestinal tissues were taken for the detection of relevant cytokines using qRT-PCR. The primers for PCR used in this paper are listed in **Table 2**.

### Detection of Antibodies in Serum, Skin and Intestinal Mucus

The blood, skin and intestinal mucus were taken from immunized rainbow trout and the supernatant was removed by filtering with a 0.22 μm membrane. The serum was inactivated at 56°C for 20 min and diluted using the 2-fold dilution method. The diluted sample was mixed with an equal volume of viral solution in which the viral value had been determined and incubated at 16°C for 1 h. 100 µL of the mixture was inoculated into the 96-well plate with well-grown cells. The 96-well plates were incubated at 16°C and 100 μL of maintenance solution (L-15 + Hapes) was added after 1 hour, observed and recorded. The efficacy of the neutralizing antibody was calculated using the criterion of protection of 50% of the cell pores from CPE.

### Splenic Lymphocyte Proliferation Assay

The rainbow trout spleen was removed under aseptic conditions and put into L-15 containing 10% penicillin-streptomycin. The tissue was passed through a 200-mesh cell sieve to obtain a cell suspension. The cell suspension was added to the percoll isolate slowly, then centrifuged and the cell bands collected between the isolates. The collected cells were washed and then 100 uL of inoculated cells were taken and placed into 96-well plate cell culture plates until they stuck to the wall. 10 μL of pPG-612-CK6-G/*L.casei* 393 were taken and added to the pining cells and incubated overnight at 37°C. Assays and calculations were performed based on the methods of the CCK-8 kit.

### Immunoprotective Assay With Recombinant *Lactobacillus*

The first oral immunization was given to rainbow trout after temporary rearing. The experimental group was orally immunized with 200 μL pPG-612-CK6-G/*L.casei* 393 suspension, the control group with an equal dose of pPG-612/*L.casei*, and the blank group with PBS. The second equal immunization dose was given on day 30 after the first immunization. On day 60 after the second immunization, rainbow trout was injected with 200 μL 10^7^ TCID50/mL of IHNV. The number of deaths was observed and recorded, and the survival curve was drawn.

### Statistical Analysis

Experimental data are presented as mean ± SD and P values were calculated using one-way ANOVA and LSD *post hoc* tests (SPSS Statistics, version 23.0, IBM, Chicago, Ill, USA). * represents *p*<0.05 (a difference), ** represents *p*<0.01 (a significant difference), and *** represents *p*<0.001 (a highly significant difference).

## Results

### Identification of pPG-612-CK6-G and pPG-612-CK6-G/*L.casei* 393

**Figure 1:**
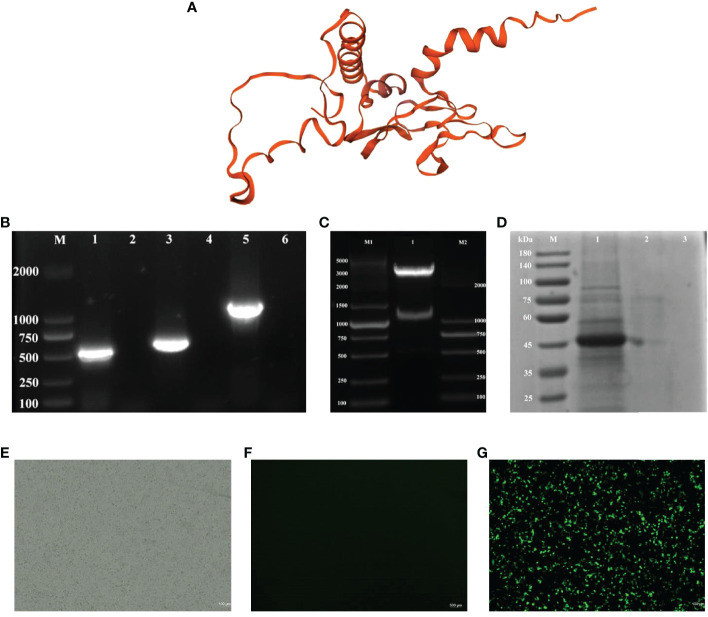
Identification of pPG-612-CK6-G*/L.casei* 393 and the protein expression. **(A)** Three-dimensional structures of recombinant CK6 and G proteins **(B)** Electrophoresis plots of amplification of different target genes, line 1: the G gene strip; line 3: the CK6 gene strip; line 5: the recombinant CK6-G strip; line 2, 4 and 6: blank control; **(C)** Double digestion electrophoresis plots of recombinant plasmids, 1: recombinant plasmid double digestion strip; M1: DNA marker DL5000, M2: DNA marker DL2000; **(D)** SDS-PAGE identification of protein expression of CK6-G gene, line 1: the pPG-612-CK6-G*/L.casei* 393 after induction; line 2: the uninduced pPG-612-CK6-G*/L.casei* 393; 3: blank control; M: 10-190 kDa protein marker **(E)** Indirect immunofluorescence map of the blank group. **(F)** Indirect immunofluorescence map of the control group. **(G)** Indirect immunofluorescence plot of the experimental group.

The description of the results in 3.1 is modified to “The results in **Figure 1A** show that the predicted tertiary structure of the protein following recombination of the CK6 and G genes. And the target gene can be obtained by PCR amplification in pPG-612-CK6-G/L.casei 393 in **Figure 1B**. The double digestion electropherogram in **Figure 1C** shows a band of about 1200 bp, suggesting that the construction of rpPG-612 is correct. The SDS-PAGE result in **Figure 1D** shows a specific reaction band at 43 kDa in the induced pPG-612/L.casei, and the non-induced pPG-612/L.casei and blank groups did not show specific bands. The **Figures 1E–G** indirect immunofluorescence results showing that CK6 and G genes can be expressed intracellularly after recombination. This indicates that the CK6-G gene in the recombinant pPG-612-CK6-G plasmid is capable of being expressed in L.casei, which provides a basis for future experiments

### Stability and Growth Curve of pPG-612-CK6-G/*L.casei* 393

**Figure 2:**
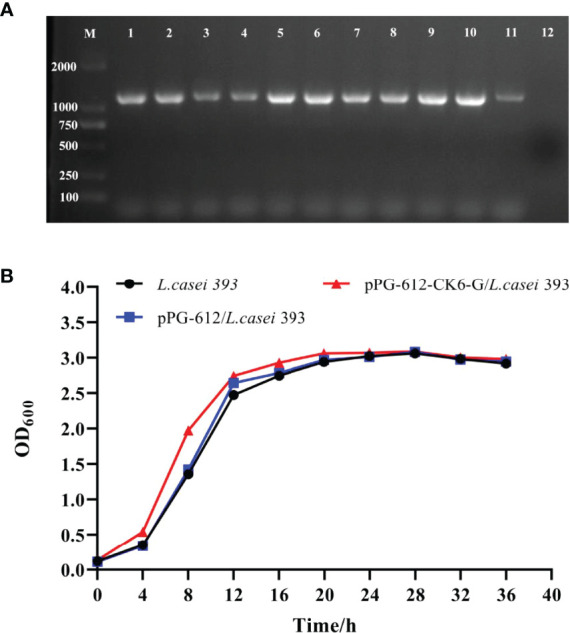
Genetic stability and growth of pPG-612-CK6-G/*L.casei* 393. **(A)** Identification of samples taken at different times for 50 consecutive generations, line 1: first generation sap; line 2: the 5th generation sap; line 3: the 10th generation sap; line 4: the 15th generation sap; line 5: the 20th generation sap; line 6: the 25th generation sap; line 7: the 30th generation sap; line 8: the 35th generation sap; line 9: the 40th generation sap; line 10: the 45th generation sap; line 11: the 50th generation sap; line 12: blank control; M: DNA marker DL2000; **(B)** Growth curves of pPG-612-CK6-G/*L.casei*, pPG-612/*L.casei* and *L.casei* over 36 h.

According to the results in **Figure 2A**, the amplification of pPG-612-CK6-G/*L.casei* 393 at different passages resulted in a target band of approximately 1200 bp in size, indicating that the constructed pPG-612-CK6-G was able to be amplified in the stable presence of *L.casei* without loss of the target gene. The results in **Figure 2B** show that the growth of pPG-612-CK6-G/*L.casei* 393 was similar to that of *L.casei* and pPG-612/*L.casei*, indicating that the inserted exogenous CK6-G protein gene did not affect the normal growth of *L.casei*, providing a basis for growth differences in subsequent experiments.

### Effect of pPG-612-CK6-G/*L.casei* 393 on Specific Antibody Production

**Figure 3:**
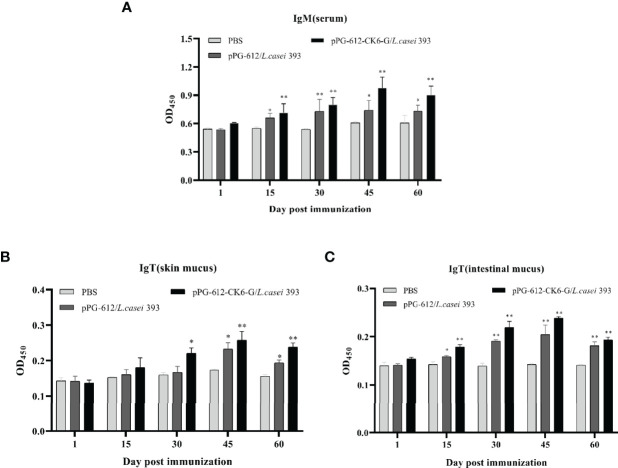
Levels of specific antibody expression in different tissues 60 days after secondary immunization. **(A)** Changes in IgM levels in serum at different time periods; **(B)** changes in IgT levels in skin mucosa at different time periods; **(C)** changes in IgT levels in intestinal mucosa at different time periods. Data are presented as mean ± SD and P values were calculated using one-way ANOVA and LSD *post hoc* tests. * represents *p*<0.05, ** represents *p*<0.01.

According to **Figure 3A** it can be seen that after oral immunization of rainbow trout with rpPG-612/*L.casei*, the specific antibody IgM in the serum reached its highest point on the 15th day after the second immunization. When specific antibody IgT was examined in the intestinal mucosa and skin mucosa, it was found that production began in the body 15 d after the first immunization and reached its highest point at day 15 after the second immunization (**Figures 3B, C**). The antibody levels in both serum and mucosa were significantly different from those in the blank and control groups. This indicates that the recombinant *L.casei* not only induced the production of IgM, but also IgT, a specific antibody against IHNV in rainbow trout serum and mucosa. This suggests that pPG-612-CK6-G/*L.casei* enhanced the immunity of rainbow trout in this study.

### Effect of pPG-612-CK6-G/*L.casei* 393 on Cytokines in Different Tissues

**Figure 4:**
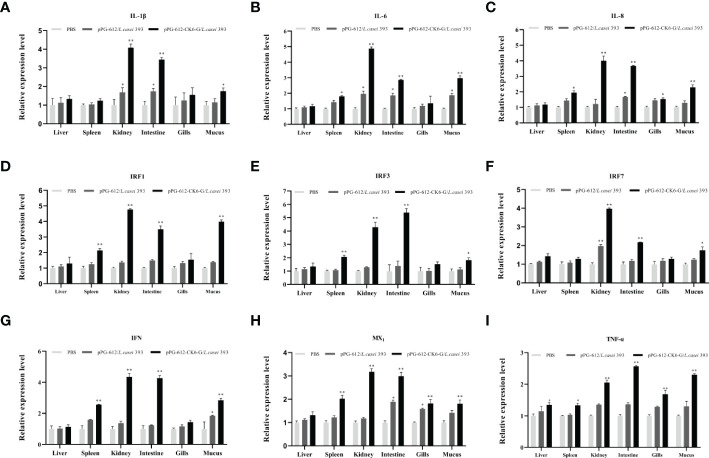
The relative expression levels of cytokines in the liver, spleen, kidney, intestine, gill, and skin mucus on the 15th day after secondary immunization. **(A)** the relative expression of *IL-1β*; **(B)** the relative expression of *IL-4*; **(C)** the relative expression of *IL-6*; **(D)** the relative expression of *IRF1*; **(E)** the relative expression of *IRF3*; **(F)** the relative expression of *IRF7*; **(G)** the relative expression of *IFN*; **(H)** the relative expression of *MX1*; **(I)** the relative expression of *TNF-α*. Data are presented as mean ± SD and P values were calculated using one-way ANOVA and LSD *post hoc* tests. * represents *p*<0.05, ** represents *p*<0.01.

The results presented in **Figures 4A–C** show significant differences in the expression of *IL-1β*, *IL-4* and *IL-8* in the kidney, intestine and skin mucus after oral pPG-612-CK6-G/*L.casei* 393 immunization compared to the control group. The results presented in **Figures 4D–G** show significant differences in the expression of *IRF1*, *IRF3*, *IRF7*, IFN in the spleen, kidney, intestine and skin mucus after oral administration of recombinant vaccine compared to the control group. In **Figure 4H**, *MX1* was significantly different from the control group in all tissues except the liver after oral administration of the recombinant vaccine. The results in **Figure 4I** show that *TNF-α* differed from the control group in all tissues after oral administration of the recombinant vaccine. This shows that the recombinant vaccine in this study can enhance the expression of inflammatory and antiviral factors in rainbow trout.

### Effect of pPG-612-CK6-G/*L.casei* 393 on Neutralizing Antibodies Against IHNV

**Figure 5:**
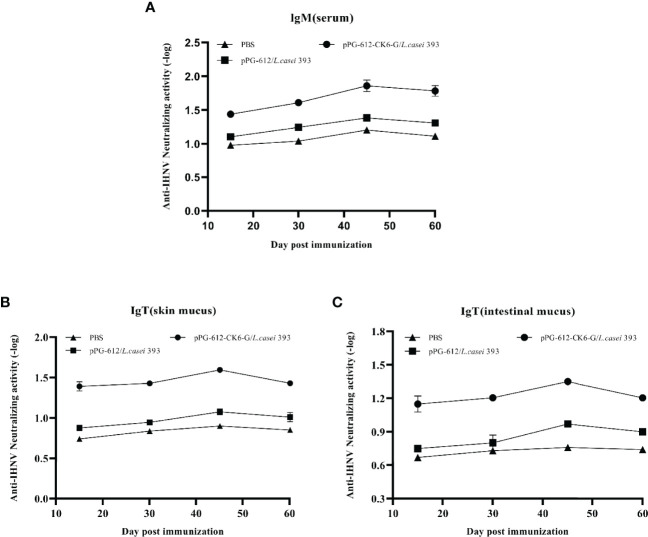
Levels of antibody-neutralizing virus in different immunized tissues at 60 days after secondary immunization. **(A)** Changes in antibody neutralization levels in serum at different time periods; **(B)** changes in antibody neutralization levels in skin mucosa at different time periods; **(C)** changes in antibody neutralization levels in intestinal mucosa at different time periods. Data are presented as mean ± SD.

As shown in **Figure 5A**, the potency of serum neutralizing antibodies increased gradually after 15 d following secondary immunization, reaching the highest antibody levels at d 15, with oral pPG-612-CK6-G/*L.casei* 393 inducing higher antibody levels than both control pPG-612/*L.casei* and PBS. The results in **Figures 5B, C** show that the ability of both the intestinal and skin mucosa to neutralize antibodies was enhanced after secondary immunization. The potency of oral pPG-612-CK6-G/*L.casei* 393 against IHNV was 10^1.86^, 10^1.59^ and 10^1.35^ in serum, skin mucosa and intestinal mucosa, respectively, as calculated by the Karber method. The results suggest that oral immunization with *L.casei* can prevent disease by stimulating the body to secrete antibodies in the serum and mucous membranes to neutralize the virus.

### Effect of pPG-612-CK6-G/*L.casei* 393 on Proliferation of Splenic Lymphocytes

**Figure 6:**
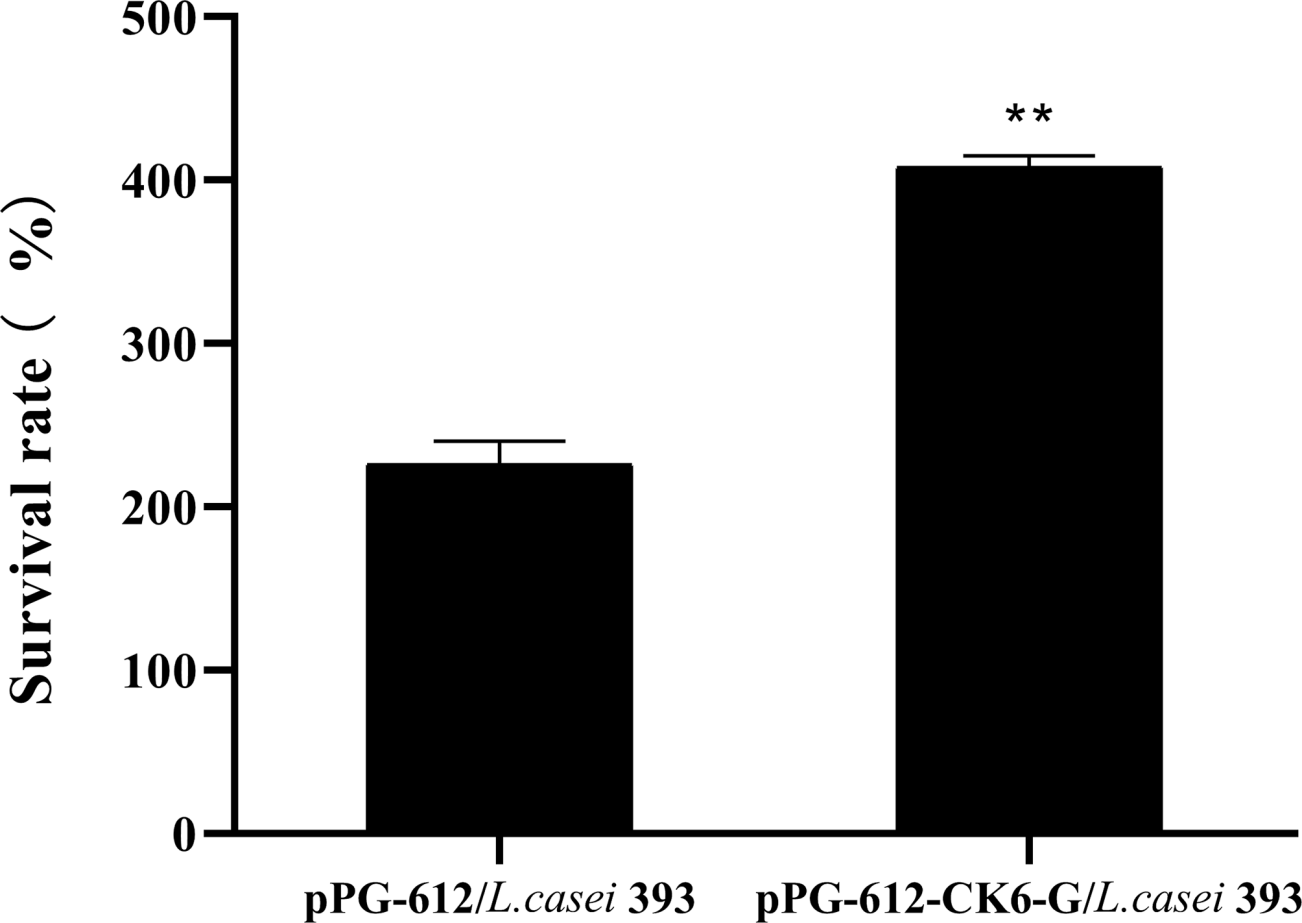
Effect of oral administration of pPG-612-CK6-G/*L.casei* 393 on lymphocyte proliferation at day 60 after secondary immunisation. Data are presented as mean ± SD and P values were calculated using one-way ANOVA and LSD *post hoc* tests. ** represents *p*<0.01.

According to the results in **Figure 6**, it can be seen that the oral administration of pPG-612-CK6-G/*L.casei* 393 leads to a significantly higher survival rate than pPG-612/*L.casei*. This indicates that the pPG-612-CK6-G/*L.casei* 393 can increase the activity of splenic lymphocytes in rainbow trout and promote the proliferation of splenic lymphocytes, which can improve the immunity of rainbow trout.

### Immunoprotective Effect of pPG-612-CK6-G/*L.casei* 393 on Rainbow Trout

**Figure 7:**
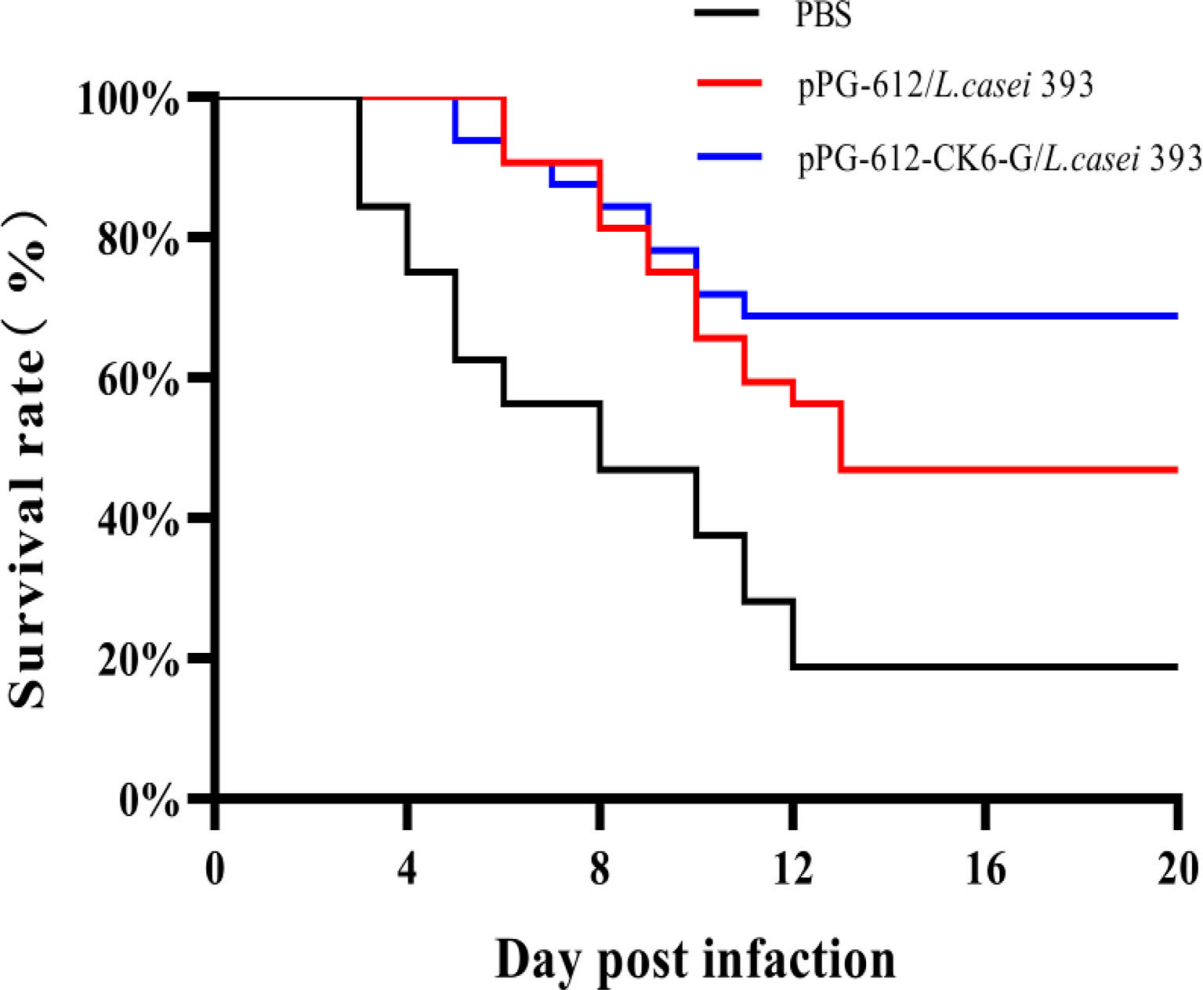
IHNV was injected on the 60th day after re-immunization, and the survival rate curve of oral pPG-612-CK6-G/*L.casei* 393 and the control group was observed within 14 days.

As shown in **Figure 7**, the rainbow trout that were treated with PBS began to die on day 3 after IHNV injection, while the deaths of rainbow trout treated with both oral pPG-612-CK6-G/*L.casei* 393 and *L.casei* were delayed. Based on the survival curves, it can be seen that the survival rate of the blank group was 10%, the oral pPG-612/*L.casei* group was 43.33%, and the oral pPG-612-CK6-G/*L.casei* 393 group was 66.67%. It can be seen that the pPG-612-CK6-G/*L.casei* 393 in this experiment has a good protective effect on rainbow trout.

## Discussion

There have been many studies on *Lactobacillus* because of its flexible expression of antigens which makes them more easily recognized by the host immune system inducing an immune response from the host. For these reasons many studies use lactic acid bacteria as a carrier for oral vaccines and to construct a lactic acid bacteria expression system (28). Pérez-Sáncheza’s found a significant increase in the expression of some cytokines in head kidney and intestine after immunization of rainbow trout with *L.casei* (29). Maqsood et al. found that the recombinant *Lactobacillus* plantarum system constructed with pPG612 as a vector for oral immunization of chickens was able to stimulate the body to produce an immune response with a significant protective effect (30). Duan et al. constructed a recombinant *L. casei* expression system to immunize rainbow trout with the VP2 protein of IPNV, and the results showed that recombinant *L.casei* induced local mucosal and systemic immune responses and reduced the ability of rainbow trout to become infected with IPNV (31). In the present study, the recombinant *L.casei* expression system constructed for immunization of rainbow trout was found to induce an immune response and produce specific antibodies in rainbow trout. The results of the attack assay showed that the survival rate of rainbow trout immunized by oral administration of *L.casei* was significantly higher than that of the control group, indicating that the constructed recombinant *Lactobacillus* system had a good protective effect on rainbow trout. The results of this study were similar to those reported in the literature, indicating that the recombinant lactic acid bacteria expression system constructed by truncating the G protein also had good protective effects.

The fish immune system includes immune organs, immune cells and immune active substances (7). The mucosal immune system is the first line of defense for fish immunity, and it is rich in mucosal-like lymphoid tissue that plays an important role in defending against pathogen invasion (32).Scleractinian fish possess a variety of immunoglobulins, such as IgM and IgT, of which IgM is mainly found in serum and IgT in mucosal tissues (33). Adachi et al. constructed recombinant *L.casei* with HPV E7 protein and found that mice immunized with these recombinant *L.casei* exhibited an effective mucosal immune response (34). It was found that oral administration of recombinant *L.casei* co-expressing the T-lymphocyte peptide 290 of the swine fever virus and the VP2 antigen of the porcine microvirus activated a mucosal response to produce IgA antibodies (35). Zhang et al. constructed secreted recombinant lactic acid bacteria from the outer membrane protein Omp W of *Aeromonas vivax* to immunize carp and found that IgM levels in the fish were higher after immunization with recombinant lactic acid bacteria than in the control group (36). Shirdast et al. introduced the Omp31 gene of *Brucella abortus* into lactococcal immunized mice and the results showed a significant increase in the levels of sIgA, IgA, IgM and IgG antibodies in the mice (37). In this study, we found that after oral immunization with pPG-612/*L.casei*, the levels of IgM and IgT in rainbow trout increased as the immunization time was delayed. The highest antibody levels were reached at d 15 after the secondary immunization, with significant differences compared to the control group. This indicates that the recombinant lactic acid bacteria in this study can increase the IgM levels of anti-IHNV in rainbow trout serum, as well as the levels of IgT in mucosal tissues. It has been shown that when viruses or bacteria infect rainbow trout, they stimulate alterations in the expression of host inflammatory factors (38). It was found that macrophages from rainbow trout can induce the expression of *IL-1β* and *TNF-α* by stimulation of LPS (39, 40). Lebre et al. found that aluminum adjuvant promotes cytokine secretion in mice after intraperitoneal injection of chitosan-aluminum salt adjuvant (41). In this study, it was found that oral administration of pPG-612-CK6-G/*L.casei* 393 increased the expression of cytokines related to interleukin, interferon and tumor necrosis factor in the host after reinfection with the virus. This suggests that the oral vaccine can stimulate the body to increase the expression of antiviral-related cytokines and thus resist viral infection.

Xu et al. found that after oral immunization with recombinant *L.casei* in mice, the number of empty cell spots decreased by 77.2%, and the ability to neutralize antibodies was stronger (42). Yu et al. constructed recombinant *L.casei* from the S protein of the transmissible gastroenteritis of swine virus (TGEV) and the S protein of the porcine epidemic diarrhea virus (PEDV) and showed that recombinant *L.casei* elevated antibody levels in mice and also promoted lymphocyte proliferation (43). Similarly, the results of the present study show that the ability of serum to neutralize antibodies and the ability of splenic lymphocytes to proliferate *in vitro* were enhanced after oral immunization of rainbow trout with recombinant *lactobacilli*. The results of this experiment were consistent with those reported in the literature, which indicated that the pPG-612-CK6-G/*L.casei* in this study had a good effect on host production of neutralizing antibodies.

In summary, this study successfully constructed a recombinant *L.casei* system expressing IHNV truncated G protein and rainbow trout chemokine CK6. The expression system has good immunogenicity and can promote the mucosal immune system of rainbow trout to respond and secrete corresponding antibodies, thus improving the immunity and survival rate of the organism. This study provided a new idea and method for the development of an IHNV vaccine.

## Data Availability Statement

The original contributions presented in the study are included in the article/supplementary material. Further inquiries can be directed to the corresponding author.

## Ethics Statement

The animal study was reviewed and approved by the Ethical Committee for Animal Experiments of Northeastern Agricultural University, China. All animal experiments complied with the guidelines of the Animal Welfare Council of China.

## Author Contributions

All authors listed have made a substantial, direct, and intellectual contribution to the work and approved it for publication. All authors contributed to the article and approved the submitted version.

## Funding

This work was supported by grants from the National Natural Science Foundation of China (No. 32173012).

## Conflict of Interest

The authors declare that the research was conducted in the absence of any commercial or financial relationships that could be construed as a potential conflict of interest.

## Publisher’s Note

All claims expressed in this article are solely those of the authors and do not necessarily represent those of their affiliated organizations, or those of the publisher, the editors and the reviewers. Any product that may be evaluated in this article, or claim that may be made by its manufacturer, is not guaranteed or endorsed by the publisher.
